# Enhancing recovery and outcomes of sternal closure in cardiac surgery: Early results of a 400-patient comparison of suture tapes and steel wires

**DOI:** 10.1016/j.xjtc.2025.03.016

**Published:** 2025-05-04

**Authors:** Ujjawal Kumar, Usman Aslam, Tyler Phillips, Zacharya Khalpey, Zain Khalpey

**Affiliations:** a Department of Cardiac Surgery, HonorHealth, Scottsdale, Ariz; b Khalpey AI Lab, Applied & Translational AI Research Institute (ATARI), Scottsdale, Ariz; c School of Clinical Medicine, University of Cambridge, Cambridge, United Kingdom; d Department of General Surgery, HonorHealth, Scottsdale, Ariz

**Keywords:** median sternotomy, suture tapes, sternal closure

## Abstract

**Background:**

Conventional steel wires may be inadequate for patients at high risk of sternal complications. We compared steel wires with a novel sternal closure system involving suture tapes, aiming to reduce sternal complications and enhance postoperative recovery, particularly in high-risk patients.

**Methods:**

A total of 400 consecutive patients undergoing cardiac surgery via median sternotomy were analyzed retrospectively. Steel wires were used for patients 1 to 200 and suture tapes were used for patients 201 to 400. Preoperative, intraoperative, and postoperative data were compared between the 2 groups of patients.

**Results:**

The 2 groups were generally similar in terms of preoperative characteristics. The suture tape group had lower rates of sternal wound infection (1% vs 5%) and sternal dehiscence (0% vs 6%). Postoperative hospital admission also was significantly shorter (7 days vs 10 days). Suture tape patients had significantly less pain at 14-day and 30-day follow-ups, with significantly lower opioid use (125 vs 175 morphine milligram equivalents).

**Conclusions:**

Suture tape sternal closure was effective, reproducible, and safe. It showed significant advantages over steel wires, including lower rates of sternal infection, dehiscence, and postoperative pain, as well as decreased opioid usage, and shorter hospital admission and closure times. We demonstrate the significant potential of this novel sternal closure system, especially for patients susceptible to sternal complications. Extended follow-up will be vital to demonstrate long-term efficacy.

## Graphical Abstract

**Figure:**
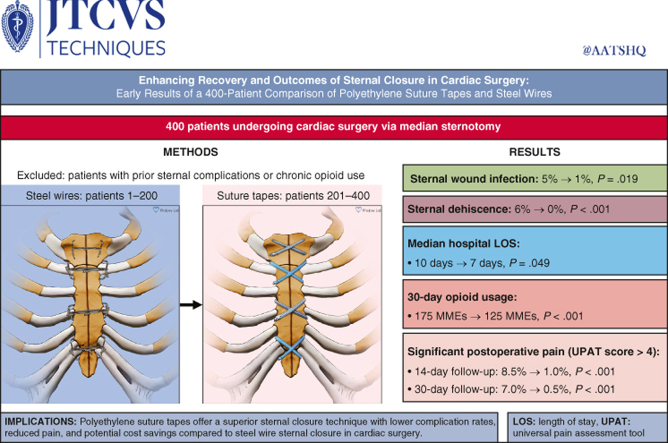

**Figure:**
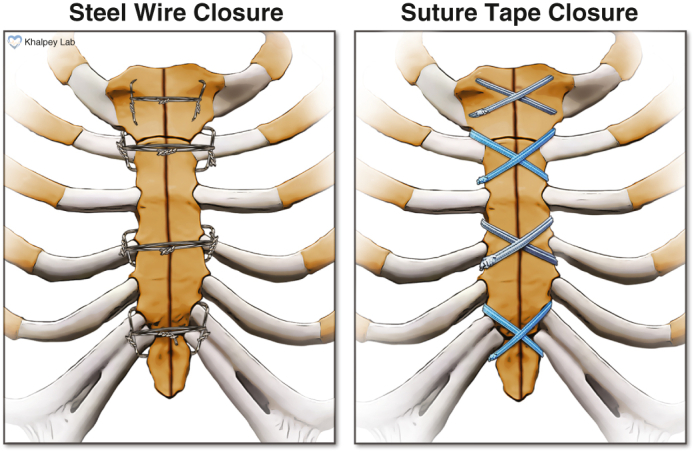
The two sternal closure approaches – steel wires and polyethylene suture tapes.

**Central Message**

Compared to steel wires, ultra-high molecular weight polyethylene suture tapes offer a superior sternal closure system with lower complication rates, reduced pain, and potential cost savings considering sternal protection adjuncts.

**Perspective**

Sternal complications after median sternotomy for cardiac surgery have significant adverse consequences. Compared to steel wires, this innovative system shows a significant reduction in sternal wound infections, dehiscence, and postoperative pain while potentially decreasing healthcare costs and improving patient outcomes.

Despite increases in percutaneous and minimally invasive approaches, higher-risk patients are now undergoing median sternotomy thanks to cardiac surgical and anesthetic improvements. However, because of either preoperative or intraoperative factors, certain patients are at significant risk of sternal wound infection or dehiscence, and conventional steel wires may be inadequate in these patients. Sternal wound dehiscence usually occurs within the first 2 postoperative weeks,^1^ with a reported mortality rate of up to 9%.^2^ Considering the significant consequences of sternal wound complications, optimizing sternal closure approaches is crucial.

Identifying patients at increased risk of postoperative sternal complications allows for targeted interventions and improves shared decision making and informed consent. Obesity is one of the most significant risk factors, increasing the mechanical stress on the sternum and the likelihood of instability, particularly after such activities as breathing and coughing. Greater mechanical stress on the sternum may compromise sternal reapproximation and cause bone cut-through, both of which can result in sternal instability and impaired healing. Notably, the risk increases with increasing body mass index.^3^ Similarly, patients with chronic obstructive pulmonary disease also are at high risk, as they are prone to bouts of frequent and forceful coughing. Additionally, frail patients have a 2-fold higher risk of sternal complications.^4^ Osteoporosis and long-term steroid therapy also compromise bone quality, increasing the risk of sternal complications. Addressing the mechanical component is important, as wound dehiscence typically occurs in the early postoperative period, before complete bone healing.^5^ The risk of sternal complications is also increased by impaired sternal and chest wall perfusion. This may occur due to microvascular disease in patients with diabetes or a smoking history^6^ and in patients undergoing either bilateral or pedicled internal mammary artery harvesting.^7^

Here we present our experiences and outcomes with the use of a novel sternal closure system, comparing it to the conventional approach that uses steel wires. Ultra-high molecular weight polyethylene suture tapes (FiberTape and TigerTape; Arthrex, Inc) are already widely used in orthopedic surgery^8^ in place of steel wires and are receiving increasing interest for sternal closure.^9^ They offer significant advantages over steel wires, with increased strength and reduced risk of bone cut-through due to their flat, broad contact patch. In ex vivo load-to-failure tests, the suture tapes withstood nearly double the force withstood by the steel wires across several configurations.^10^^,^^11^ Additionally, steel has the potential for increased corrosion due to friction when implanted, potentially leading to connective tissue encapsulation and increased risk of postoperative infection and bone instability.^12^ This risk is eliminated when using a sternal closure system that does not involve steel. The increasing complexity of cardiac surgical patients calls for improved sternal closure systems, and the present study aimed to evaluate a novel system and its feasibility for regular use.

## Patients and Methods

**Figure 1:**
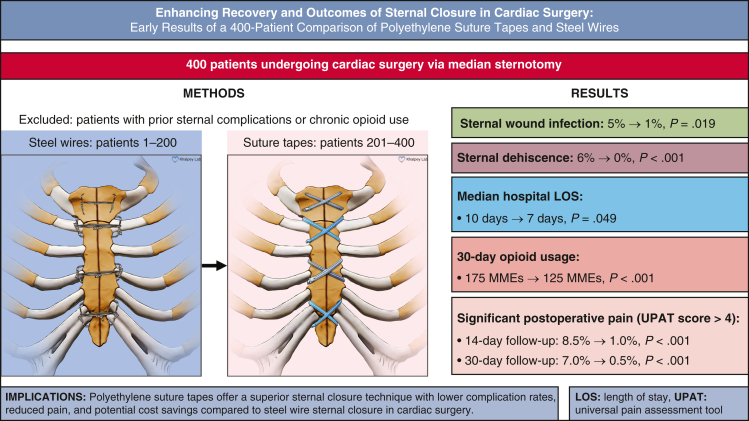
Graphical abstract: a comparison of sternal closure techniques and their outcomes.

This retrospective study included 400 consecutive patients who underwent median sternotomy for cardiac surgery (isolated coronary artery bypass grafting [CABG], isolated valve surgery, combined CABG and valve surgery, or major aortic surgery) performed by a single surgeon. Patients 1 to 200 (June 2022 to April 2023) were the last 200 patients who underwent sternal closure using sternal wires, and patients 201 to 400 (April 2023 to May 2024) were the first 200 patients to undergo sternal closure using the new suture tape system. Patients with a history of sternal complications after median sternotomy were excluded, as were patients with a history of chronic opioid use. Patients under the age of 18 years were also excluded. All patients consented to the relevant surgical procedures. The study received Institutional Review Board approval (#23-0025; approved March 26, 2023), with the requirement for specific patient consent waived, as information was recorded such that the identity of the human subjects cannot be readily ascertained directly or through identifiers linked to the subjects. Figure 1 provides a graphical abstract of the study.

### Data Collection

Demographic data, preoperative and operative characteristics, and postoperative outcomes were collected from the institutional electronic health record^13^ and verified by external adjudicators for registry contribution. Preoperative data collected included STS risk scores (mortality and deep sternal wound infection), as well as risk factors for sternal complications (obesity, diabetes, previous sternotomy, smoking history, chronic obstructive pulmonary disease, and immunosuppressive medication use). Data on other comorbidities were collected as well.

Established criteria were used to define heart failure^14^ and chronic kidney disease.^15^ Sternal wound infection and dehiscence were defined using the Society of Thoracic Surgeons criteria for entry into the Adult Cardiac Surgery Database,^16^ with infection being either superficial or deep. Superficial infections involved skin, subcutaneous tissue, and/or pectoralis fascia, with erythema, purulent discharge, tenderness, and positive wound cultures. Deep infections also had bony involvement with positive mediastinal cultures and evidence of mediastinitis, as well as such features as chest pain, sternal instability, fever, and purulent drainage from the mediastinum.

### Operative Technique

**Figure 2:**
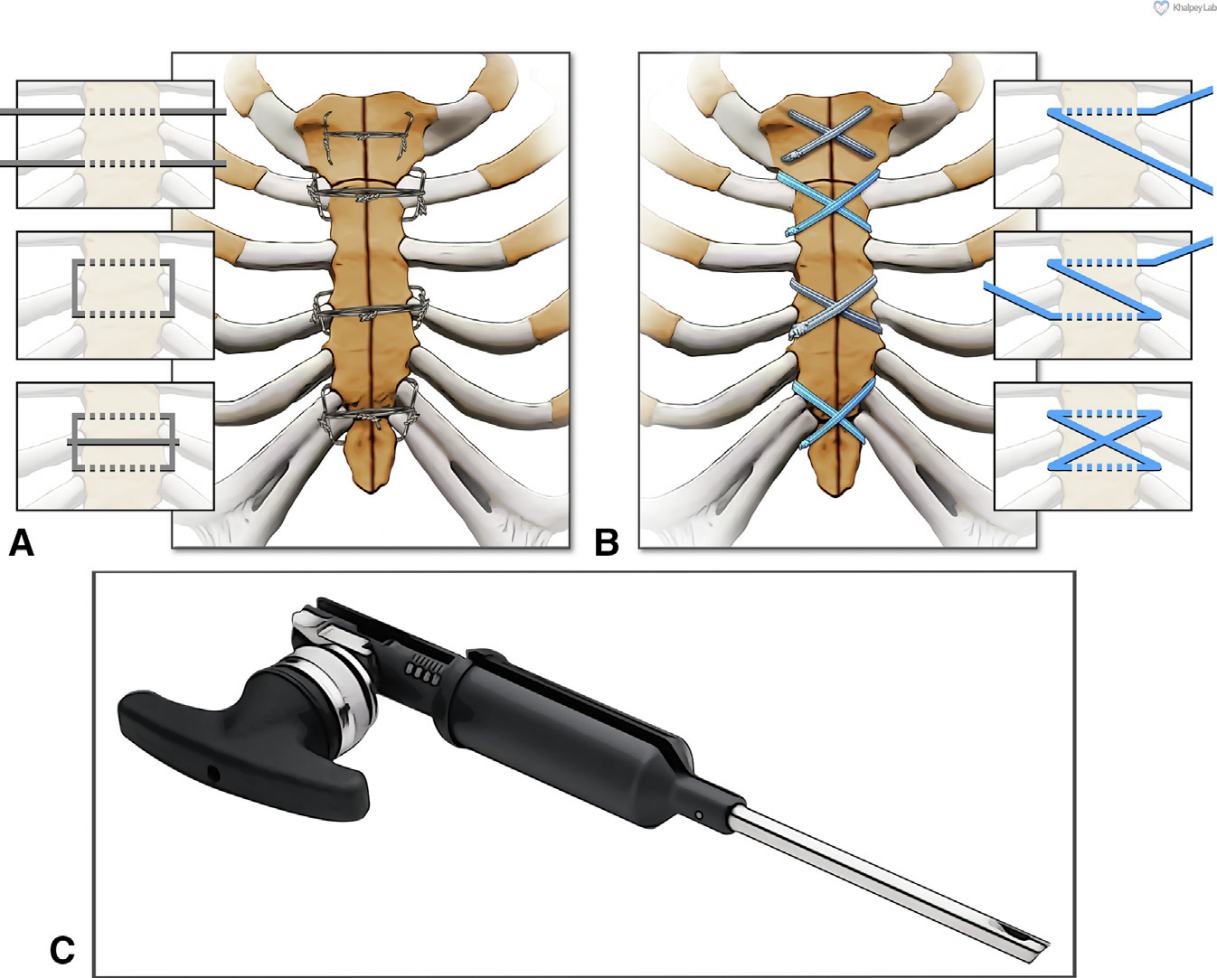
Sternal closure techniques used in this study. A, Steel wires used in a semi-Robicsek cerclage configuration. B, Suture tapes used in a figure-of-eight configuration. C, A tensioner used to apply a precise amount of force on the suture tapes once placed.

Once the main aspects of the surgical procedure were completed and hemostasis was confirmed, attention was turned to sternal closure, which was performed by the attending surgeon in all cases. The sternal closure technique differed between the 2 study groups, with all other operative techniques being identical. In study patients 1 to 200, sternal closure was achieved using 8 individual steel wires as described previously (Figure 2, *A*).^17^ In patients 201 to 400, however, ultra-high molecular weight polyethylene suture tapes (FiberTape and TigerTape) were used for sternal closure after being presoaked in 0.4% vancomycin solution (1 g/250 cc sterile saline) for 5 minutes. The tapes were placed in a figure-of-eight configuration (Figure 2, *B*) and tightened with a precise amount of force using a tensioner (Figure 2, *C*), as described previously.^17^ After tightening, 2 half-hitch locking knots were tied, and the excess tape was cut. Following sternal reapproximation and verification of sternal stability, thorough wound lavage was performed with 0.4% vancomycin solution, and vancomycin paste (prepared by reconstituting 4 g of vancomycin powder with 10 cc of sterile saline) was applied for all patients. Subcutaneous tissues were closed using 0, 2-0, and 4-0 polydioxanone sutures (Stratafix PDS; Ethicon), and the skin was closed with 4-0 poliglecaprone subcuticular suture (Stratafix Monocryl; Ethicon) and Dermabond/Prineo (Ethicon).

### Follow-up and Outcomes

Postoperative outcomes recorded included total intensive care unit and hospital length of stay, hospital mortality, sternal wound infection, and dehiscence. Patients were reviewed in the follow-up clinic as standard practice at 2 weeks and 1 month postoperatively. At each visit, a thorough wound inspection was performed. As a key patient-reported outcome measure (PROM), postoperative pain was assessed using a patient-reported numeric rating scale (NRS-11) as part of the Universal Pain Assessment Tool (UPAT).^18^ This scale assesses the patient's ability to perform their necessary activities of daily living (ADL) and thus was felt to be the most useful measure of the significance of any postoperative pain and its impact on functional status. Postoperative pain was deemed significant if the pain score exceeded 4 (indicating significant ADL limitation). Appropriate analgesia was ensured for all patients with significant pain at follow-up.

### Statistical Analysis

Normally distributed data are presented as mean ± standard deviation, and the Student's *t*-test was used for comparing groups. Nonparametric data are presented as a median and interquartile range, with the Kruskal-Wallis test used to compare groups. Categorical variables are presented as number (%), and groups were compared using the χ^2^ or Fisher exact test if the expected frequency was less than 5. All statistical analyses were performed using R version 4.4.1.^19^

## Results

### Preoperative Patient Characteristics

As shown in Tables 1 and 2, groups had generally similar preoperative characteristics. Rates of most sternal risk factors were similar in the 2 study groups, but diabetes mellitus (35.0% vs 24.0%; *P* = .021) and a positive smoking history (42.5% vs 28.5%; *P* = .005) were more common in the suture tape group. Operative risk (based on Society of Thoracic Surgeons score) was comparable in the 2 groups.

**Table 1:** Relevant risk factors for postoperative sternal complications

Variable	Wires	Suture tapes	*P* value
Number	200	200	N/A
Body mass index kg/m^2^, mean ± SD	33.28 ± 8.94	32.09 ± 8.61	.237
STS DSWI score, median (IQR)	0.16 (0.10-0.27)	0.18 (0.12-0.27)	.873
Obesity, n (%)	112 (56.0)	115 (57.5)	.840
Diabetes mellitus, n (%)	48 (24.0)	70 (35.0)	.021
Previous sternotomy, n (%)	5 (2.5)	7 (3.5)	.508
Smoking history, n (%)	57 (28.5)	85 (42.5)	.005
COPD, n (%)	10 (5.0)	12 (6.0)	.826
Immunosuppressive medications, n (%)	17 (8.5)	20 (10.0)	.730

*N/A*, Not applicable; *STS*, Society of Thoracic Surgeons; *DSWI*, deep sternal wound infection; *IQR*, interquartile range; *COPD*, chronic obstructive pulmonary disease.

**Table 2:** Other preoperative characteristics and comorbidities

Variable	Wires	Suture tapes	*P* value
Number	200	200	N/A
Age, y, mean ± SD	66.62 ± 10.77	65.84 ± 11.46	.516
Male sex, n (%)	148 (74.0)	150 (75.0)	.909
STS score, median (IQR)	1.32 (0.78-2.41)	1.48 (0.98-2.05)	.665
Hypertension, n (%)	147 (73.5)	151 (75.5)	.731
Hyperlipidemia, n (%)	149 (74.5)	147 (73.5)	.909
Coronary artery disease, n (%)	120 (60.0)	115 (57.5)	.684
Obstructive sleep apnea, n (%)	44 (22.0)	41 (20.5)	.807
Heart failure, n (%)	115 (57.5)	105 (52.5)	.366
Chronic kidney disease, n (%)	38 (19.0)	47 (23.5)	.328
Prior atrial fibrillation, n (%)	33 (16.5)	21 (10.5)	.107
Prior myocardial infarction, n (%)	32 (16.0)	25 (12.5)	.391
Prior PCI, n (%)	31 (15.5)	29 (14.5)	.889
Prior stroke, n (%)	15 (7.5)	8 (4.0)	.198
CHA_2_DS_2_-VASc score, median (IQR)	3 (2-4)	3 (1-3)	.582
HAS-BLED score, median (IQR)	2 (1-3)	2 (1-2)	.259

*N/A*, Not applicable; *STS*, Society of Thoracic Surgeons; *IQR*, interquartile range; *PCI*, percutaneous coronary intervention.

### Operative Characteristics

**Figure 3:**
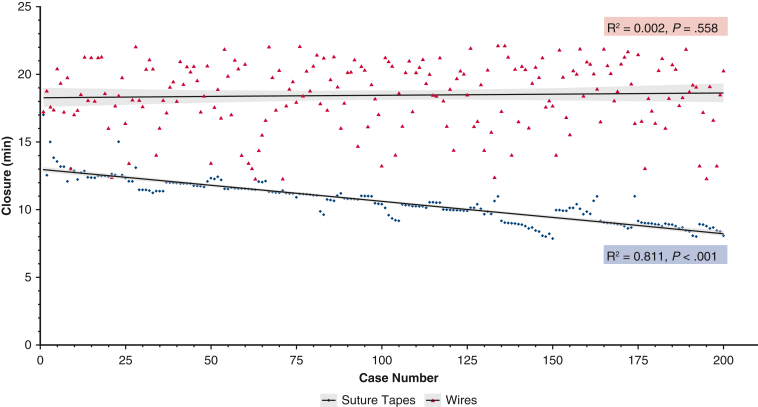
Consecutive closure times for the 2 study groups. Closure times in the suture tape group generally decreased with consecutive patients, with minimal variation from the trend. However, the wire closure group showed no overall trend, with significant variation.

Considering operative characteristics (Table 3), the 2 groups were comparable in terms of procedural urgency and type, as well as cardiopulmonary bypass and aortic cross-clamp times. Crucially, in all patients undergoing CABG using the left internal mammary artery, the conduit was harvested in a skeletonized fashion to better preserve sternal perfusion. Closure time was significantly shorter in the suture tape group than in the wire group (11.10 minutes vs 18.69 minutes; *P* < .001). As shown in Figure 3, closure time was decreased significantly with consecutive patients in the suture tape group (*R*^2^ = 0.811; *P* < .001). Conversely, no significant trend was seen in the wire group (*R*^2^ = 0.002; *P* = .558), with significant variation.

**Table 3:** Operative characteristics

Variable	Wires	Suture tapes	*P* value
Number	200	200	N/A
Elective, n (%)	163 (81.5)	148 (74.0)	.092
Procedure type, n (%)			.281
CABG ± Maze	106 (53.0)	99 (49.5)	
Valve ± Maze	54 (27.0)	64 (32)	
CABG and valve ± Maze	12 (6.0)	17 (8.5)	
Aortic	19 (9.5)	17 (8.5)	
Other	9 (4.5)	3 (1.5)	
LIMA skeletonization	101 (100.0)	100 (100.0)	>.999
Cardiopulmonary bypass (min)	99 ± 51	90 ± 39	.091
Aortic cross-clamp (min)	66 ± 31	68 ± 27	.532
Closure (min)	18.69 ± 2.36	11.10 ± 1.40	<.001

*N/A*, Not applicable; *CABG*, coronary artery bypass grafting; *LIMA*, left internal mammary artery.

### Postoperative Outcomes

Postoperative outcomes are shown in Table 4. Comparing groups, the suture tape group generally showed superior outcomes, with significantly lower rates of sternal wound infection (1.0% vs 5.0%; *P* = .019) and sternal wound dehiscence (0.0% vs 6.0%; *P* < .001). In the suture group, fewer patients experienced significant pain (as defined previously) at 14 days (1.0% vs 8.5%; *P* < .001) and 30 days (0.5% vs 7.0%; *P* < .001). Postoperative opioid use was significantly lower in the suture tape group (125 vs 175 morphine milligram equivalents; *P* < .001). This reduction in sternal wound complications was supported by multivariable regression analysis (Table 5).

**Table 4:** Postoperative outcomes

Variable	Wires	Suture tapes	*P* value
Number	200	200	N/A
ICU LOS, d, median (IQR)	3 (2-4)	2 (2-4)	.698
Hospital LOS, d, median (IQR)	10 (5-17)	7 (5-11)	.049
30-d opioid use (MME), median (IQR)	175 (138-225)	125 (62-188)	<.001
Hospital mortality, n (%)	6 (3.0)	4 (2.0)	.522
Sternal wound infection, n (%)	10 (5.0)	2 (1.0)	.019
Sternal wound dehiscence, n (%)	12 (6.0)	0 (0.0)	<.001
Significant pain at 14 d, n (%)	17 (8.5)	2 (1.0)	<.001
Significant pain at 30 d, n (%)	14 (7.0)	1 (0.5)	<.001

*N/A*, Not applicable; *ICU*, intensive care unit; *LOS*, length of stay; *IQR*, interquartile range; *MME*, morphine milligram equivalent.

**Table 5:** Multivariable regression

Variable	Infection		Dehiscence		Pain: 14 d		Pain: 30 d	
	OR	*P* value	OR	*P* value	OR	*P* value	OR	*P* value
Suture tapes	0.14	.014	0.00	.992	0.12	.005	0.08	.017
Diabetes	6.32	.004	4.98	.009	1.14	.814	0.85	.808
Smoking history	1.43	.562	1.09	.893	0.41	.167	0.00	.990

*OR*, Odds ratio.

Intensive care unit and hospital length of stay were similar in the 2 groups. In addition, the suture tape group did not require the use of additional chest stabilization and wound protection adjuncts, such as negative-pressure dressings or chest binders/support vests. These adjuncts were previously used for all high-risk patients (diabetics, obese patients, or those who had undergone surgery using the left internal mammary artery) but were quickly phased out in the suture tape group as they were found to be unnecessary.

## Discussion

This study demonstrates that this novel suture tape system for sternal closure is safe and superior to conventional steel wire closure in our early clinical experience. Given the prevalence of risk factors for postoperative sternal complications in these patients,^20^ optimizing the sternal closure technique is particularly relevant. The suture tape group had excellent outcomes with only 2 sternal wound infections (both superficial). The steel wire group had significantly higher infection rates, including a greater incidence of deep wound infections, which are associated with worse prognoses than superficial infections.

Notably, the superficial infections in the suture tape group both resolved with a short course of antibiotics, whereas the deep infections in the wire group frequently required surgical intervention (eg, debridement, reconstruction), which are associated with poorer outcomes.^21^ These findings are particularly significant given the vulnerability of our patient population; 35.0% of the study population had diabetes, and 42.5% had a history of smoking, both of which are well-established risk factors for sternal wound complications.

Another key finding was the marked reduction in postoperative pain in the suture tape group. Postoperative pain is a major barrier to recovery and rehabilitation,^22^^,^^23^ and thus strategies to reduce pain are highly beneficial in improving both short-term and long-term outcomes. At 14 days, only 1.0% of the suture tape patients reported significant pain limiting daily activities, decreasing to 0.5% at 30 days. The steel wire group had substantially higher rates of pain, 8.5% at 14 days and 7.0% at 30 days.

Reductions in postoperative pain also are likely to reduce the use of opioid analgesic medications, which are independently associated with cardiovascular impairment and poor cardiac outcomes^24^ and have the potential for dependence and misuse if used long-term.^25^^,^^26^ Published literature has shown that approximately 50% of patients have moderate to severe chronic pain after cardiac surgery,^27^ usually related to the sternotomy. This leads to relatively high long-term opioid use, with approximately 10% of opioid-naive patients (such as those included in this study) still using opioids at 90 days after cardiac surgery.^28^ We found that this reduction in pain was supported by a 29% lower rate of opioid use in the suture tape group.

In this study, pain was assessed using the UPAT, with a focus on its impact on ADL, a critical PROM. Pain was deemed significant if it interfered with ADL, as indicated by an UPAT NRS-11 score >4. This approach allowed for an objective comparison of postoperative pain between the groups while accounting for its functional impact. The suture tape group's markedly lower rates of significant pain reflect a clinically meaningful improvement not only in comfort, but also in the ability to resume ADL during recovery. PROMs are being increasingly recognized as valuable clinical outcome measures, providing insight into patients' quality of life and functional recovery. Moreover, PROMs are key to shared decision making, aiding the selection of therapeutic options based on patient priorities.

Operatively, the suture tape system reduced mean closure time from 18.69 minutes to 11.10 minutes (*P* < .001). Notably, closure time decreased significantly with consecutive patients in the suture tape group (*R*^2^ = 0.811; *P* < .001), indicating a rapid learning curve and consistent technique. In contrast, the wire group showed no significant trend, as the conventional closure technique and exhibited considerable variability. An unexpected but significant benefit was the safe elimination of chest stabilization adjuncts (support vests, negative pressure dressings) in the suture tape group, which were previously required for high-risk patients (eg, diabetes, obesity).

Our results align with and expand on previous research, which has demonstrated the efficacy of suture tapes in thoracic transplantation^9^ and veterinary surgery.^29^ The reproducibility of positive outcomes across different contexts supports further evaluation and adoption of this system.

### Benefits of This Novel Sternal Closure System

This novel sternal closure system has key advantages over steel wires for sternal closure. The biomechanical advantage is supported by previous ex vivo studies demonstrating that polyethylene suture tapes can withstand nearly double the force of steel wires across various configurations.^10^^,^^30^ This greater strength is likely responsible for the increased sternal stability seen in the suture tape group, with resulting lower rates of sternal wound dehiscence postoperatively. Additionally, the use of the tensioner for the suture tapes results in a consistent, precise amount of force, explaining the reproducible nature of the closure time, with minimal adjustments needed after the suture tapes were tensioned. Conversely, the sternal wires often required adjusting and retensioning, hence the significant variation in closure time.

This pain reduction likely stems from the suture tapes' broader, flatter profile, which may reduce localized tissue pressure and damage compared to traditional steel wires. The flatter, soft knot stack compared to steel wires also likely contributed to reduced pain postoperatively. This different footprint on the sternal bone also reduces the risk of bone cut-through, protecting sternal integrity, particularly in patients with compromised bone quality. Additionally, the flat footprint allows for additional rigid fixation systems on top of the suture tapes if these are required in extremely high-risk patients. Presoaking the suture tapes in vancomycin solution also might have contributed to the low infection rates. Additionally, the material properties of the suture tapes appear to create an environment less conducive to bacterial colonization than steel wires. Finally, if resternotomy is necessary, the suture tapes can be cut easily with heavy scissors without the need for wire cutters or specialized instruments.

### Health Economics Considerations

Although a comprehensive cost-effectiveness study is in progress, initial financial assessments indicate that the suture tape system delivers substantial economic advantages alongside its clinical benefits. The higher upfront costs for suture tape closure (4 × $100 per suture tape, 8 × $20 per sternal wire at current prices) may be financially justified when considering total costs, particularly for high-risk patients. Costs may be reduced through shorter operative times and the lack of additional protective devices (support vests and negative pressure dressings) that are required with wire closure. The lower complication rates may improve quality metrics, potentially enhancing hospital reimbursement. Additionally, reduced complications and less postoperative pain could translate to shorter hospital stays, driving further cost reductions.

### Study Limitations

Although the results are encouraging, this study has certain limitations. While the 2 groups were well-matched, the study's single-surgeon retrospective nature introduces potential bias and may limit the generalizability. Multicenter randomized studies as well as extended follow-up are needed to validate our findings. Also prudent would be a detailed cost-effectiveness comparison of sternal closure methods, in particular examining potential cost reductions from fewer complications and shortened hospital stays. Future price changes associated with these technologies also should be considered.

## Conclusions

Suture tape sternal closure offers significant advantages over conventional steel wire closure. We found significantly lower rates of sternal wound infections, dehiscence, and postoperative pain. Operatively, the suture tape system significantly reduced closure time, with a clear learning curve. The safe elimination of sternal adjuncts led to substantial cost savings, particularly in high-risk patients. Longer-term follow-up has suggested improved patient outcomes and potential healthcare cost reductions. The system's superior biomechanical properties, reproducible technique, and consistent performance make it a promising innovation for optimizing cardiac surgical care, especially in high-risk populations.

## Conflict of Interest Statement

Dr Zain Khalpey has received personal consulting fees from and has taught at CME events sponsored by Arthrex Inc. All other authors reported no conflicts of interest.

The *Journal* policy requires editors and reviewers to disclose conflicts of interest and to decline handling or reviewing manuscripts for which they may have a conflict of interest. The editors and reviewers of this article have no conflicts of interest.

## References

[1] Wilkinson G.A., Clarke D.B. (1988). Median sternotomy dehiscence: a modified wire suture closure technique. Eur J Cardiothorac Surg.

[2] Losanoff J.E., Collier A.D., Wagner-Mann C.C. (2004). Biomechanical comparison of median sternotomy closures. Ann Thorac Surg.

[3] Molina J.E., Lew R.S.L., Hyland K.J. (2004). Postoperative sternal dehiscence in obese patients: incidence and prevention. Ann Thorac Surg.

[4] Lee J.A., Yanagawa B., An K.R. (2021). Frailty and pre-frailty in cardiac surgery: a systematic review and meta-analysis of 66,448 patients. J Cardiothorac Surg.

[5] Shih C.C., Shih C.M., Su Y.Y., Lin S.J. (2004). Potential risk of sternal wires. Eur J Cardiothorac Surg.

[6] Schimmer C., Reents W., Berneder S. (2008). Prevention of sternal dehiscence and infection in high-risk patients: a prospective randomized multicenter trial. Ann Thorac Surg.

[7] Cheng K., Rehman S.M., Taggart D.P. (2015). A review of differing techniques of mammary artery harvesting on sternal perfusion: time for a randomized study?. Ann Thorac Surg.

[8] Deranlot J., Maurel N., Diop A. (2014). Abrasive properties of braided polyblend sutures in cuff tendon repair: an in vitro biomechanical study exploring regular and tape sutures. Arthroscopy.

[9] Coster J.N., Chan E.G., Furukawa M., Sanchez P.G. (2022). Experience using a flexible reinforced fiber suture for sternal closure in bilateral lung transplantation recipients undergoing bilateral transverse thoracosternotomy. JTCVS Tech.

[10] Arthrex Inc (2021). FiberTape® sternal closure. Data on file (APT 5056). Naples, FL. https://www.arthrex.com/resources/FL1-000439-en-US/fibertape-sternal-closure?referringteam=shoulder.

[11] Hägerich L.M., Dyrna F.G.E., Katthagen J.C. (2022). Cerclage performance analysis – a biomechanical comparison of different techniques and materials. BMC Musculoskelet Disord.

[12] Thomsen M., Thomas P. (2017). [Compatibility and allergies of osteosynthesis materials]. Unfallchirurg.

[13] Epic Systems Corporation Epic hyperspace. https://www.epic.com/.

[14] Yancy C.W., Jessup M., Bozkurt B. (2013). 2013 ACCF/AHA guideline for the management of heart failure: a report of the American College of Cardiology Foundation/American Heart Association Task Force on Practice Guidelines. J Am Coll Cardiol.

[15] Kidney Disease: Improving Global Outcomes (KDIGO) CKD-MBD Update Work Group (2017). KDIGO 2017 Clinical practice guideline update for the diagnosis, evaluation, prevention, and treatment of chronic kidney disease–mineral & bone disorder (CKD-MBD). Kidney Int Suppl (2011).

[16] Society of Thoracic Surgeons (Published online January 31, 2020). STS adult cardiac surgery database data specifications. https://www.sts.org/sites/default/files/ACSD_DataSpecificationsV4.20.pdf.

[17] Khalpey Z., Kumar U.A., Aslam U. (2025). Improving sternal closure outcomes in cardiac surgery: polyethylene suture tapes vs steel wires. J Clin Med.

[18] Hayward M. (1994). Pain: clinical manual for nursing practice. Nurs Stand.

[19] R Core Team (2024). R: a language and environment for statistical computing. R package version 4.4.1. R Foundation for Statistical Computing. https://www.R-project.org/.

[20] Olbrecht V.A., Barreiro C.J., Bonde P.N. (2006). Clinical outcomes of noninfectious sternal dehiscence after median sternotomy. Ann Thorac Surg.

[21] Jonkers D., Elenbaas T., Terporten P., Nieman F., Stobberingh E. (2003). Prevalence of 90-days postoperative wound infections after cardiac surgery. Eur J Cardiothorac Surg.

[22] Makkad B., Heinke T.L., Sheriffdeen R. (2025). Practice advisory for postoperative pain management of cardiac surgical patients: executive summary. A report from the Society of Cardiovascular Anesthesiologists. J Cardiothorac Vasc Anesth.

[23] Huang A.P.S., Sakata R.K. (2016). Pain after sternotomy—review. Braz J Anesthesiol.

[24] Kanaya N., Murray P.A., Damron D.S. (1998). Morphine decreases cardiac contractility through a Kappa opioid receptor-mediated reduction in myofilament calcium sensitivity in rats. Anesth Analg.

[25] Waljee J.F., Li L., Brummett C.M., Englesbe M.J. (2017). Iatrogenic opioid dependence in the United States: are surgeons the gatekeepers?. Ann Surg.

[26] Kwanten L.E., O’Brien B., Anwar S. (2019). Opioid-based anesthesia and analgesia for adult cardiac surgery: history and narrative review of the literature. J Cardiothorac Vasc Anesth.

[27] Ochroch J., Usman A., Kiefer J. (2021). Reducing opioid use in patients undergoing cardiac surgery—preoperative, intraoperative, and critical care strategies. J Cardiothorac Vasc Anesth.

[28] Brown C.R., Chen Z., Khurshan F., Groeneveld P.W., Desai N.D. (2020). Development of persistent opioid use after cardiac surgery. JAMA Cardiol.

[29] Rivenburg R.E., Maxwell E.A., Bertran J., Souza C.H.D., Smith B.L. (2023). Biomechanical comparison of canine median sternotomy closure using suture tape and orthopedic wire cerclage. Vet Surg.

[30] Wähnert D., Lenz M., Schlegel U., Perren S., Windolf M. (2011). Cerclage handling for improved fracture treatment. A biomechanical study on the twisting procedure. Acta Chir Orthop Traumatol Cech.

